# Synthesis Optimization of Chitosan-Derived Carbon Dots for Gelatin Capsule Waste/Gum Arabic Active Packaging Films

**DOI:** 10.3390/ijms27219886

**Published:** 2026-11-04

**Authors:** Songporn Pipatvoratam, Prangruthai Boorapha, Khanittha Chinarak, Pudthaya Kumnerdsiri, Lalitphan Kitsanayanyong, Anurak Uchuwittayakul, Jaksuma Pongsetkul, Samart Sai-ut, Saroat Rawdkuen, Passakorn Kingwascharapong

**Affiliations:** 1 Department of Fishery Products, Faculty of Fisheries, Kasetsart University, Bangkok 10900, Thailandkhanittha.ch@ku.th (K.C.); pudthaya.ku@ku.th (P.K.); ffislhk@ku.ac.th (L.K.); 2 Department of Aquaculture, Faculty of Fisheries, Kasetsart University, Bangkok 10900, Thailand; ffisarb@ku.ac.th; 3 School of Animal Technology and Innovation, Institute of Agricultural Technology, Suranaree University of Technology, Nakhon Ratchasima 30000, Thailand; jaksuma@sut.ac.th; 4 Department of Food Science, Faculty of Science, Burapha University, Chonburi 20131, Thailand; samarts@go.buu.ac.th; 5 Unit of Innovative Food Packaging and Biomaterials, School of Agro-Industry, Mae Fah Luang University, Chiang Rai 57100, Thailand; saroat@mfu.ac.th

**Keywords:** carbon dots, chitosan, hydrothermal synthesis, antioxidant activity, antimicrobial activity, bioactive film

## Abstract

This study aimed to optimize the synthesis of chitosan-derived carbon dots (CS-CDs) and incorporate them into gelatin capsule waste/Gum Arabic (Gel/GA) composite films for sustainable active packaging of minced fish. CS-CDs were synthesized hydrothermally at 180, 200, and 220 °C for 4, 5, and 6 h. The optimal condition was 200 °C for 6 h, producing spherical nanoparticles below 10 nm with strong blue fluorescence, a maximum absorbance at 293 nm, and antioxidant activities of 56% (2,2-diphenyl-1-picrylhydrazyl: DPPH) and 66% (2,2′-azino-bis(3-ethylbenzothiazoline-6-sulfonic acid): ABTS) at a concentration of 20% (*v*/*v*), together with antibacterial activity against *S. aureus*, *S. enteritidis*, and *E. coli*. The optimized CS-CDs were incorporated into Gel/GA films at 0–2% (*w*/*v*), significantly increasing tensile strength from 2.20 to 4.19 MPa and improving water resistance (*p* < 0.05). Antioxidant and antimicrobial activities increased with CS-CD concentration. Gel/GA/CD-2 exhibited the best overall performance and effectively preserved minced fish at room temperature for up to 48 h by reducing microbial growth and lipid oxidation. Overall, integrating CS-CDs with upcycled gelatin capsule waste provides a promising approach for developing multifunctional biodegradable active packaging with enhanced mechanical, antioxidant and antimicrobial properties for seafood preservation.

## 1. Introduction

Maintaining food safety and quality during processing, transportation, and storage is an important concern in the food industry. Microbial contamination and chemical changes during these stages can adversely affect food quality and reduce its shelf life. Active packaging has therefore been developed as an alternative approach to conventional packaging, with the incorporation of functional materials into packaging matrices to inhibit microbial growth, delay oxidative deterioration, and help maintain food quality during storage [1]. Recently, carbon dots (CDs) have emerged as promising nanomaterials for active food packaging applications due to their excellent optical properties, low toxicity, high water solubility, biocompatibility, facile synthesis, and the availability of diverse precursor materials [2,3,4]. In addition, the presence of surface defects and unpaired electrons endows CDs with free radical scavenging activity, while their photoelectric and photothermal characteristics contribute to antimicrobial performance. These unique features make CDs highly attractive for improving the functionality of food packaging films, particularly in terms of antioxidant and antibacterial activities, photostability, and overall packaging performance [5].

Carbon dots can generally be synthesized through two major approaches, namely top-down and bottom-up methods. Top-down strategies involve breaking down bulk carbon materials into nanoscale particles using techniques such as laser ablation, electrochemical oxidation of graphite, electrochemical treatment of multiwalled carbon nanotubes, and chemical oxidation of activated carbon. In contrast, bottom-up approaches construct CDs from molecular precursors through processes such as hydrothermal treatment, microwave irradiation, and ultrasonication [6]. Among these methods, hydrothermal synthesis has become one of the most widely employed techniques for producing biomass-derived CDs because of its operational simplicity, cost-effectiveness, and environmentally friendly nature [3,4]. During hydrothermal synthesis, aqueous precursor solutions are exposed to elevated temperatures for several hours, promoting dehydration, polymerization, and carbonization reactions that ultimately lead to the formation of CDs [3]. Importantly, hydrothermal conditions, especially reaction temperature and time, have a significant impact on the physicochemical and functional characteristics of the resultant CDs [4,7]. The formation and development of carbon dots (CDs) are strongly influenced by hydrothermal temperature and reaction time, which regulate their growth, surface chemistry, and extent of carbonization. Higher temperatures facilitate the decomposition and carbonization of precursors, thereby affecting the structural evolution of the carbon core as well as the distribution and abundance of surface functional groups [4]. Therefore, optimizing the hydrothermal conditions is important for producing CDs with the desired physicochemical and functional properties. However, the optimal synthesis conditions may vary depending on the precursor used, as differences in its chemical composition and structure can affect the carbonization and nucleation processes. Accordingly, the hydrothermal temperature and reaction time should be systematically optimized to better understand their effects on the properties of precursor-derived CDs and to determine the conditions that yield CDs with suitable characteristics for specific applications. In addition to hydrothermal conditions, the choice of precursor is an important factor in determining the properties of CDs. Although a wide range of carbon-rich biomass materials have been explored, the complex composition of some biomass precursors can lead to heterogeneous products and increase the need for purification. Consequently, obtaining consistent control over the structure and surface chemistry of CDs can be challenging when using structurally complex biomass materials [3].

Chitosan (CS), a natural biopolymer, is biodegradable, widely available, and has low toxicity and antibacterial activity. It has attracted significant attention as a precursor for carbon dot (CD) synthesis because it is abundant, inexpensive, renewable, and rich in nitrogen [8]. The amino and hydroxyl groups of chitosan facilitate the formation of nitrogen self-doped CDs with surface functional groups such as -NH_2_, -OH, and -COOH. Nitrogen incorporation can introduce additional surface defects and thereby modify the optical and electronic properties of CDs. In particular, nitrogen doping has been reported to enhance photoluminescence and electron transfer, which has been attributed to the strong electron-donating nature of nitrogen atoms [9]. Riahi et al. [10] reported that chitosan-derived CDs exhibited strong antioxidant activity, antibacterial activity against *Escherichia coli* and *Listeria monocytogenes*, and antifungal activity against *Aspergillus niger* and *Penicillium chrysogenum*. Hydrothermal treatment has also been shown to enhance the antioxidant activity and water solubility of chitosan [11]. These functional properties make chitosan-derived CDs promising candidates for active packaging applications. Nevertheless, safety remains a concern for food-contact applications. Chitosan-derived CDs showed low cytotoxicity toward L929 mouse fibroblast cells at concentrations up to 500 μg/mL in a previous in vitro study [10]. However, such findings cannot be extrapolated to chronic or repeated exposure, to other cell types, or to other exposure routes, and the long-term safety of chitosan-derived CDs remains insufficiently characterized. In addition, the properties of chitosan-derived CDs are likely to vary with hydrothermal temperature and reaction time, because these parameters influence dehydration, condensation, carbonization, nucleation, and surface functionalization, and thereby the particle size, surface functional groups, and bioactivities of the resulting CDs [9]. To our knowledge, however, the combined effects of hydrothermal temperature and time on the properties and bioactivities of CDs derived from chitosan alone have not been systematically examined, and suitable synthesis conditions for CDs intended for active packaging remain unclear. Therefore, this study aimed to screen the effects of hydrothermal temperature (180, 200, and 220 °C) and time (4, 5, and 6 h) on the characteristics and bioactivities of chitosan-derived CDs (CS-CDs) in a 3 × 3 design (nine conditions), and to select the condition with the highest antioxidant activity in both DPPH and ABTS assays, with fluorescence intensity considered as a supporting characteristic. The CS-CDs obtained under the selected condition (200 °C, 6 h) were then incorporated into Gelatin/Gum Arabic composite films, and the resulting films were evaluated for their properties and for their ability to retard spoilage of minced fish under an accelerated spoilage challenge at room temperature.

## 2. Results and Discussion

### 2.1. Characterization Studies of CS-CDs

#### 2.1.1. Optical Properties of CS-CDs

**Figure 1:**
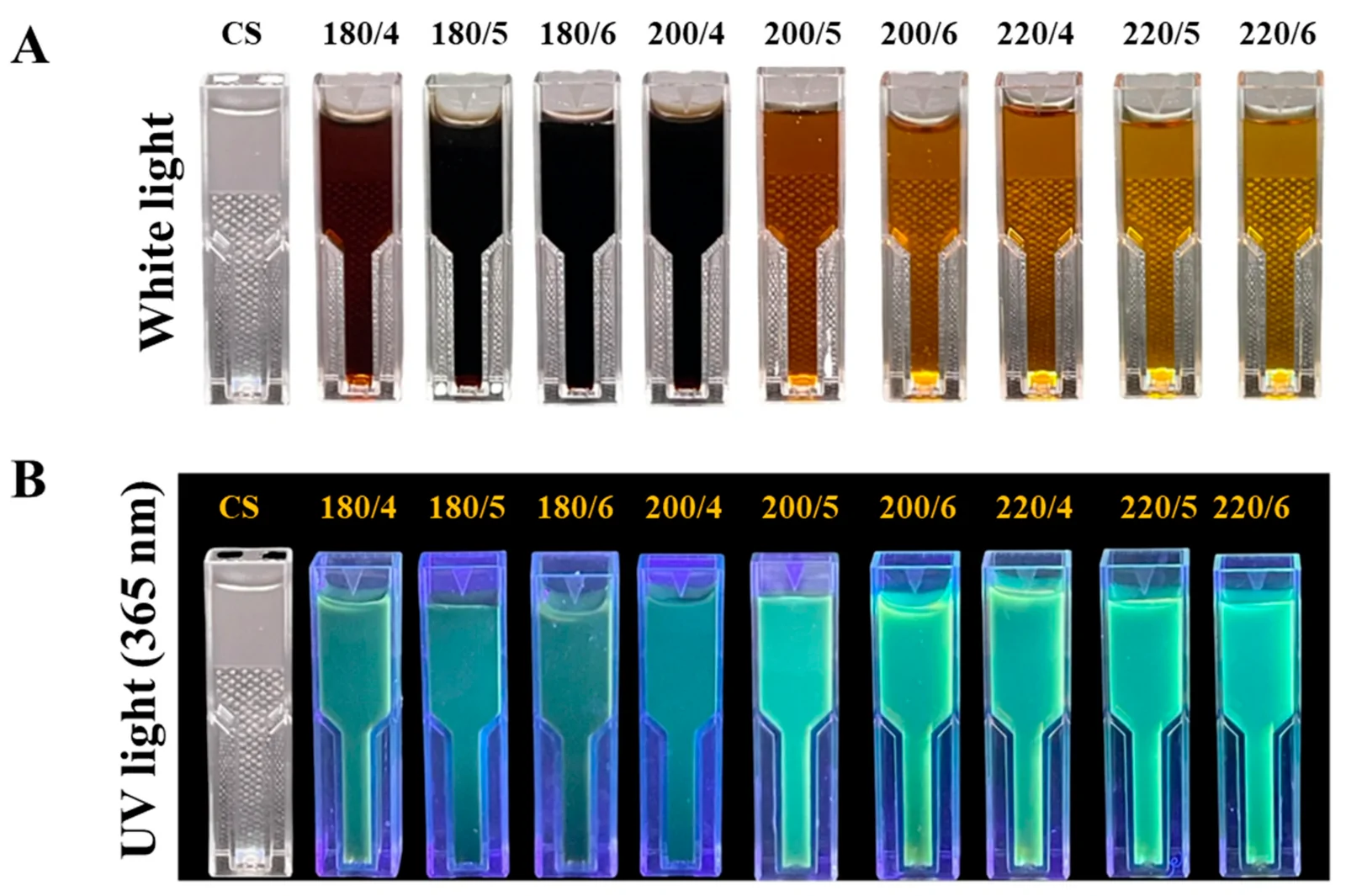
Visual confirmation of CDs under daylight (**A**) and UV light (**B**) at different synthesis temperatures and reaction times.

**Figure 2:**
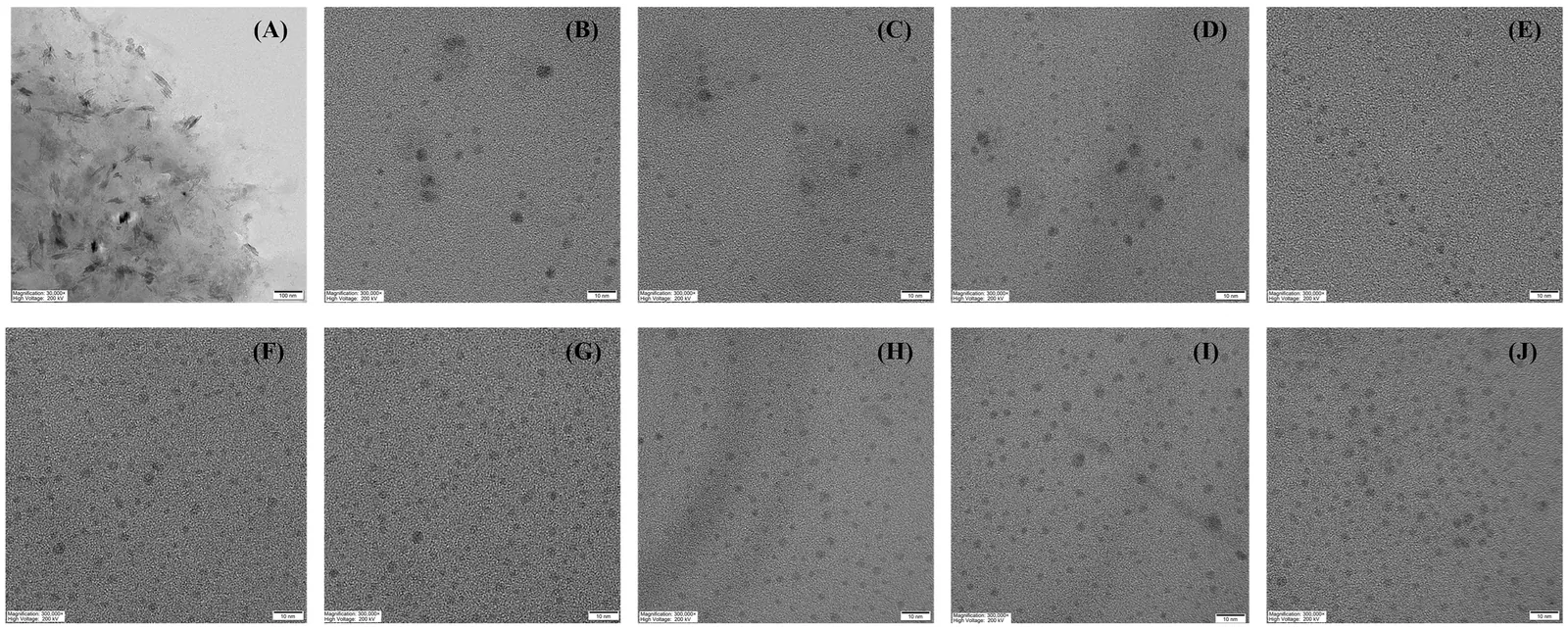
Morphology of chitosan and chitosan-derived carbon dots with different times and temperatures observed under transmission electron microscopy at 300,000× magnification (scale bar = 10 nm). (**A**) Chitosan, (**B**) 180 °C for 4 h, (**C**) 180 °C for 5 h, (**D**) 180 °C for 6 h, (**E**) 200 °C for 4 h, (**F**) 200 °C for 5 h, (**G**) 200 °C for 6 h, (**H**) 220 °C for 4 h, (**I**) 220 °C for 5 h, (**J**) 220 °C for 6 h.

**Figure 5:**
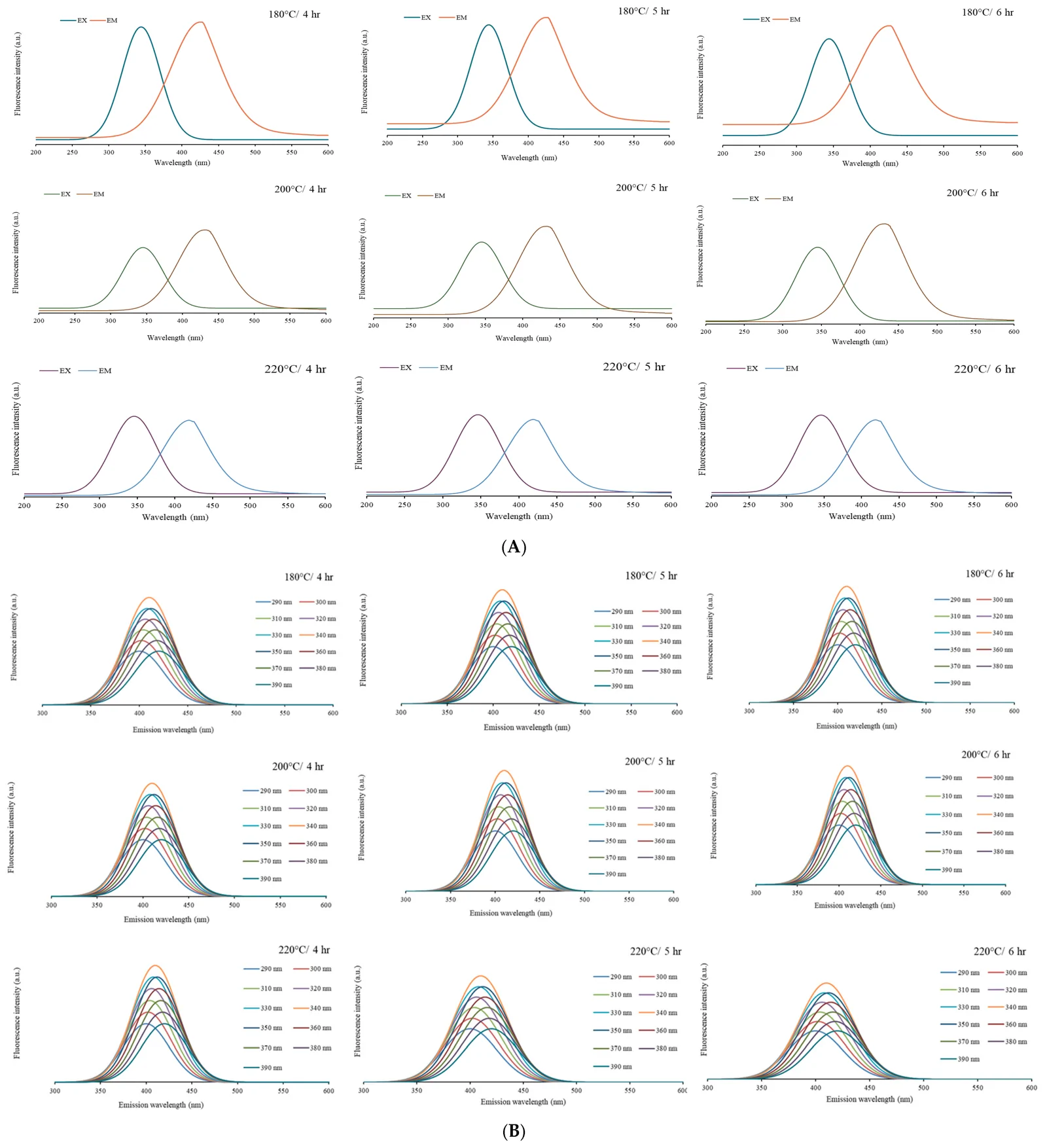
Fluorescence excitation and emission spectra (**A**) and fluorescence response at different excitation wavelengths (**B**) of carbon dots derived from chitosan synthesized at 180 °C for 4–6 h, 200 °C for 4–6 h, and 220 °C for 4–6 h.

The CS-CD solutions synthesized under varying hydrothermal temperatures and reaction times appeared as homogeneous brown liquids under visible light (Figure 1A). In contrast to the colorless CS precursor, the CS-CDs exhibited a distinct brown coloration, consistent with their ability to emit light brown fluorescence under daylight conditions. Notably, samples prepared at 180 °C showed a progressive darkening of the brown color as the reaction time increased from 4 to 6 h. Conversely, CS-CDs synthesized at higher temperatures (200 and 220 °C) exhibited a lighter brown appearance with prolonged reaction time. Under ultraviolet irradiation (365 nm), all CS-CD samples emitted blue fluorescence, as illustrated in Figure 1B. However, the fluorescence intensity and color varied depending on the synthesis conditions. Specifically, CS-CDs produced at 180 °C and 200 °C for 4 h exhibited noticeably weaker and lighter blue emission compared to those synthesized at higher temperatures or longer durations. This finding is consistent with previous reports on distillers’ grains-derived carbon dots, where the CDs exhibited progressively stronger (darker) blue fluorescence with increasing reaction temperature [4]. The observed blue fluorescence is characteristic of carbon dots (CDs) and is primarily attributed to their unique optical properties. Blue emission in carbon dots has been attributed to several mechanisms, including quantum confinement, surface states, molecular fluorophores, and sp^2^-conjugated domains [12]. The CS-CDs showed an excitation maximum at approximately 340 nm and emission maxima in the range of approximately 415–430 nm depending on the synthesis conditions (Figure 5A). The emission maximum remained nearly unchanged when the excitation wavelength was varied from 290 to 390 nm, whereas the highest intensity was obtained at an excitation of 340 nm (Figure 5B). This nearly excitation-independent behavior suggests a relatively uniform distribution of emissive states [12]. Together with the small size observed by TEM (<10 nm, Figure 2), this may be consistent with size-related or molecular-fluorophore-related emission; however, the specific mechanism was not determined in this study.

#### 2.1.2. Transmission Electron Microscope (TEM)

Transmission electron microscopy (TEM) analysis was conducted to examine the morphology and size distribution of the CS-CDs, providing direct visualization of their nanoscale structure. As shown in Figure 2, the TEM images reveal that the CS-CDs are predominantly spherical and well-dispersed, with a size distribution in the nanoscale range (<10 nm). The particle size of CS-CDs was influenced by the synthesis temperature. Specifically, CS-CDs prepared at 180 °C exhibited sizes ranging from 3.12 to 6.25 nm, whereas those synthesized at 200 and 220 °C showed smaller sizes in the range of 1.88 to 3.12 nm. These results indicate that increasing the hydrothermal temperature leads to the formation of smaller-sized carbon dots, likely due to enhanced carbonization and nucleation processes at elevated temperatures. These results are consistent with the study by Luan, Li, Lin, Lin, Zhu, Feng, Sun, Wu and Yang [5], who reported that increasing the synthesis temperature led to the formation of well-dispersed carbon dots. In addition, Wu, Mu, Luo, Zhang, Huang, Peng and Tao [4] demonstrated that particle size gradually decreased with increasing hydrothermal temperature. These variations in particle size significantly influence the optical and physicochemical properties of CDs. In general, smaller carbon dots exhibit stronger quantum confinement effects, resulting in blue-shifted fluorescence emission and enhanced surface reactivity. Such properties are advantageous for applications in sensing and bioimaging [13]. Under UV light (365 nm), the CS-CD dispersions synthesized at 200 °C for 5–6 h and at 220 °C appeared visibly brighter than those prepared at 180 °C and at 200 °C for 4 h (Figure 1B). This visual observation is qualitative and is consistent with the fluorescence spectra (Figure 5). These results highlight that variations in particle size significantly influence the optical properties of the CDs. Therefore, the observed differences in particle size among the CS-CDs underscore the critical role of synthesis parameters such as temperature, pH, and reaction time in controlling the formation of ultra-small, uniformly dispersed carbon dots [14].

#### 2.1.3. UV–Vis Spectrophotometer

**Figure 3:**
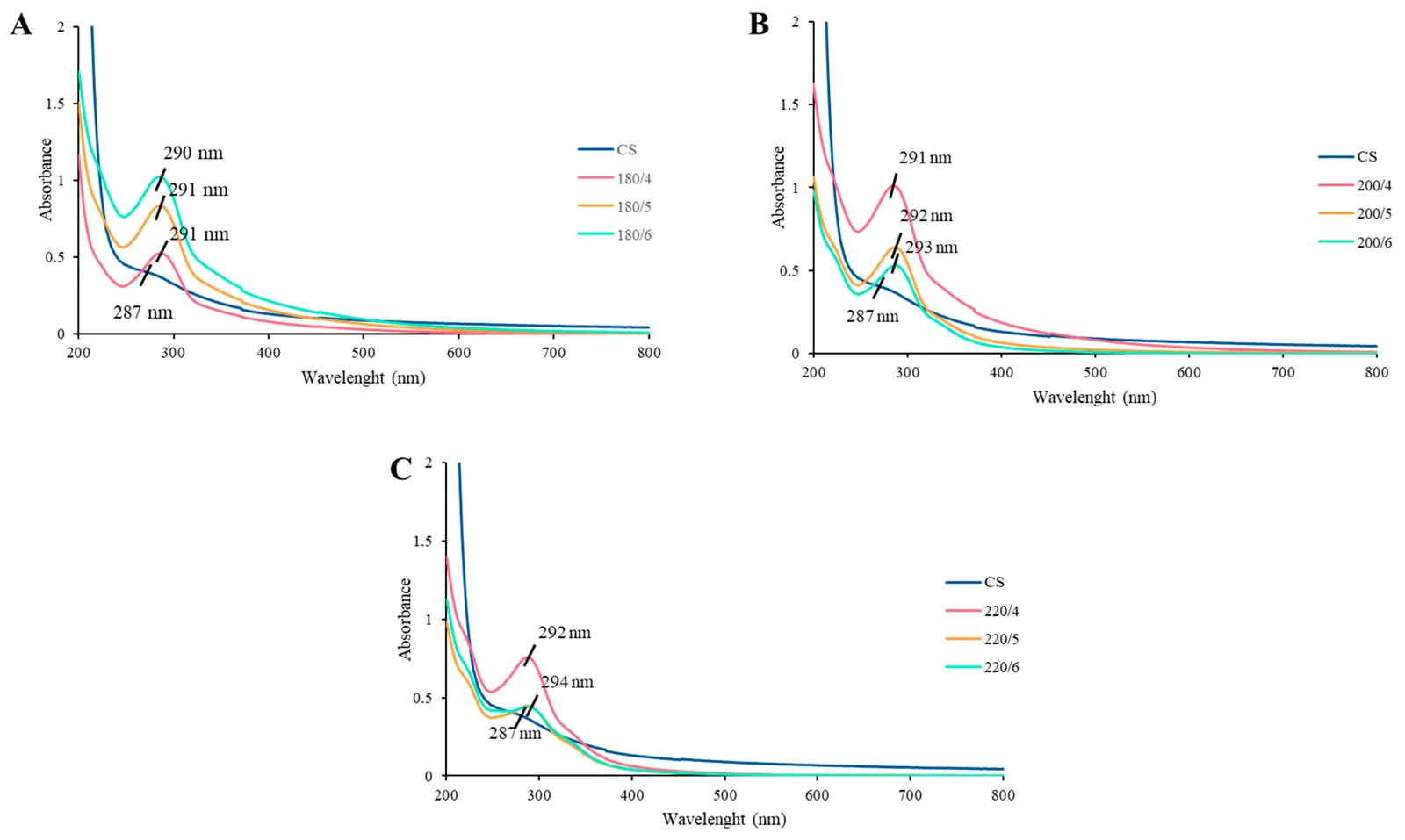
UV–Vis absorption spectra of carbon dots derived from chitosan synthesized at 180 °C for 4–6 h (**A**), 200 °C for 4–6 h (**B**), and 220 °C for 4–6 h (**C**).

UV–Vis absorption spectroscopy was employed to investigate the optical characteristics of CDs synthesized under different hydrothermal temperatures and reaction times, as shown in Figure 3. The spectrum of the CS precursor exhibited a characteristic absorption peak at 287 nm, whereas the CS-CDs displayed a slightly red-shifted peak at approximately 290 nm, which gradually shifted to 294 nm with increasing reaction temperature. This redshift suggests structural and surface modifications of the CDs during the hydrothermal process. These findings are consistent with previous reports on CDs derived from waste tea bags, which also demonstrated a temperature-dependent redshift in the absorption peak [15]. Typically, CDs exhibit strong absorption in the ultraviolet region (230–320 nm), with a tail extending into the visible region. The observed absorption behavior is commonly attributed to π–π* transitions of sp^2^-hybridized C=C bonds, indicating the presence of conjugated domains, as well as n–π* transitions associated with carbonyl (C=O) groups on the CD surface [4,16]. Furthermore, previous studies have suggested that surface passivation by different functional groups can shift the absorption toward longer wavelengths, contributing to tunable optical properties and the emergence of multi-emission behavior [16]. Therefore, the progressive redshift observed in this study may be associated with increased surface functionalization and enhanced conjugation at higher synthesis temperatures.

**Figure 4:**
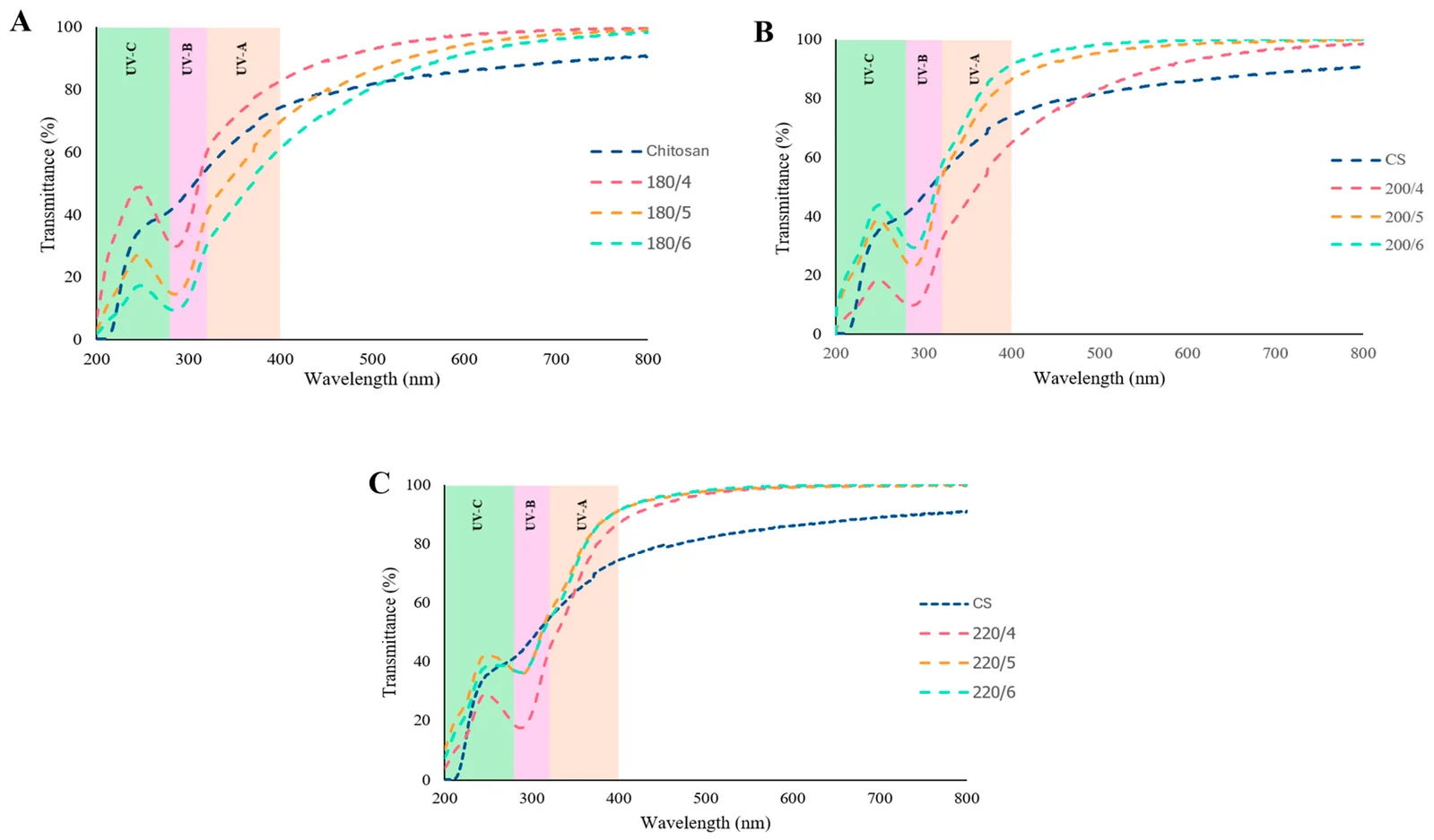
UV–visible light transmittance spectra of carbon dots derived from chitosan synthesized at 180 °C for 4–6 h (**A**), 200 °C for 4–6 h (**B**), and 220 °C for 4–6 h (**C**).

The UV absorption of CS-CD solutions synthesized under different hydrothermal conditions was evaluated (Figure 4). All samples showed high transmittance in the visible region (400–800 nm) and markedly lower transmittance in the UV region (200–400 nm). Transmittance was lower in the UV-B range (280–315 nm) than in the UV-A range (315–400 nm) and increased progressively toward 400 nm, indicating that the CS-CDs absorbed UV-B more strongly than UV-A. This absorption may be attributed to π–π* transitions of conjugated C=C structures and n–π* transitions of C=O/C=N groups in the CDs [17]. The optical absorption of CDs depends on synthesis conditions, which affect the electronic structure and surface states [16]; the underlying mechanism was not examined in this study. These observations are qualitative, based on the plotted spectra, and reflect the properties of the CDs in solution. Overall, the UV absorption of CS-CDs, together with their derivation from a renewable biopolymer, suggests that they may be of interest as bio-based UV-absorbing additives for food packaging. Their performance in films, their effect on UV-induced oxidation in food, and their safety for food-contact use remain to be investigated.

#### 2.1.4. Fluorescence Spectroscopy

The photoluminescence spectra of CS-CDs synthesized under different hydrothermal conditions are presented in Figure 5. CS-CDs prepared at 180 °C exhibited an optimal excitation wavelength at 345 nm with a maximum emission peak at 435 nm. In contrast, samples synthesized at 200 and 220 °C showed a slight redshift, with optimal excitation at 350 nm and maximum emission at 440 nm (Figure 5A). All CS-CDs displayed symmetrical emission profiles and excitation-dependent fluorescence behavior. The excitation-dependent emission characteristics suggest that the photoluminescence properties of the CS-CDs are strongly influenced by variations in particle size and surface chemical states [4]. This behavior is consistent with previous studies, which reported that higher synthesis temperatures promote the decomposition and reorganization of chitosan-derived precursors, leading to the formation of smaller carbon nanoparticles with enhanced fluorescence properties [4].

In addition, the emission spectra exhibited a typical excitation wavelength-dependent response, with a progressive redshift (bathochromic shift) as the excitation wavelength increased from 290 to 390 nm (Figure 5B). The maximum fluorescence intensity was observed at approximately 415–430 nm under an excitation wavelength of 340 nm. This tunable emission behavior is commonly attributed to the presence of multiple emissive sites and heterogeneous surface states, as well as size-dependent electronic structures within the CDs [1]. Overall, the observed excitation-dependent photoluminescence and tunable emission properties highlight the versatility of CS-CDs for potential applications in sensing, imaging, and active food packaging systems.

#### 2.1.5. Fourier Transform Infrared (FTIR) Spectroscopy

**Figure 6:**
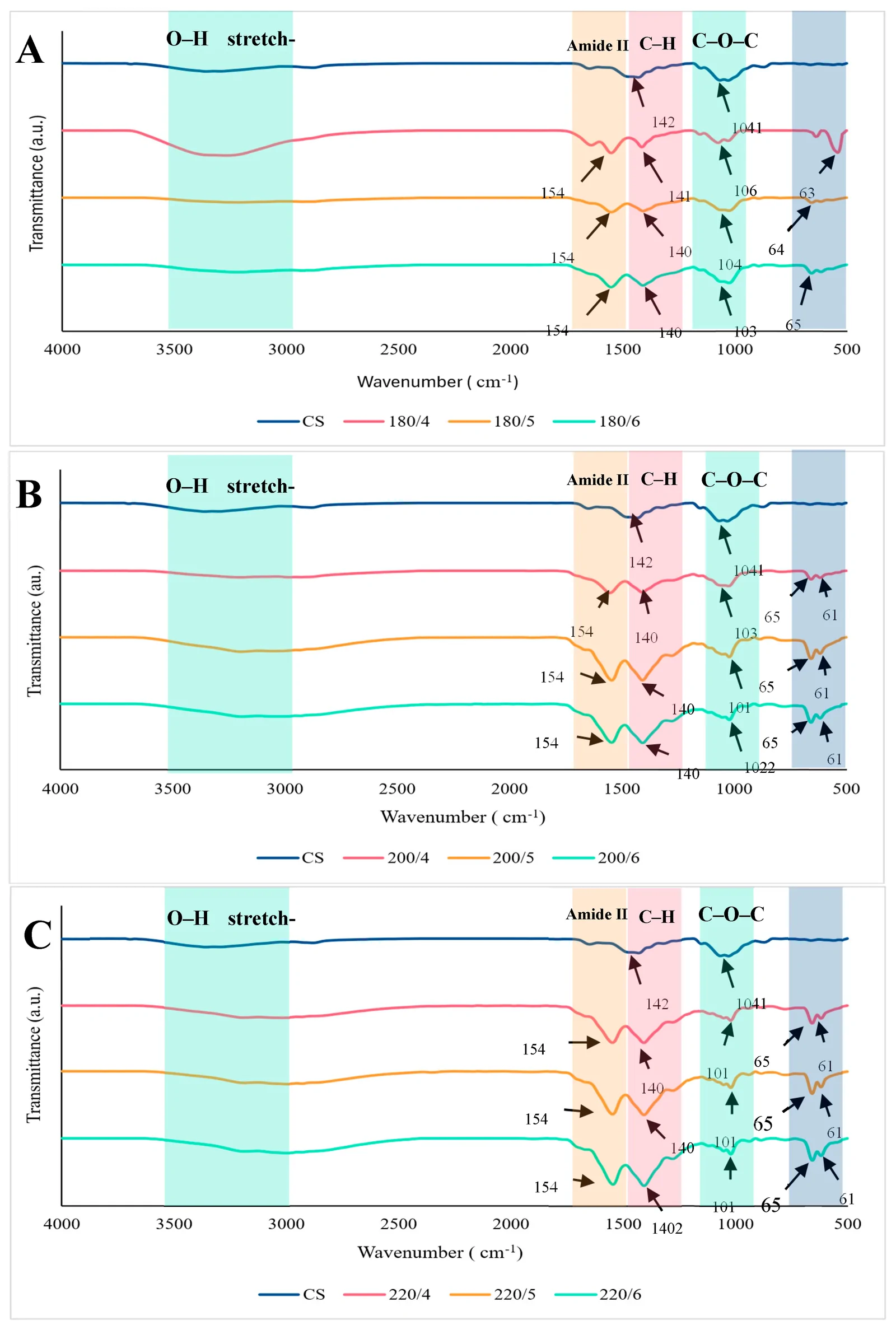
FTIR spectra of carbon dots derived from chitosan synthesized at 180 °C for 4–6 h (**A**), 200 °C for 4–6 h (**B**), and 220 °C for 4–6 h (**C**).

The chemical transformations occurring during the hydrothermal process were monitored using FTIR spectroscopy to identify the functional groups present on the surface of the CS-CDs. As presented in Figure 6, a broad absorption band observed in the range of 3000–3500 cm^−1^, corresponding to O-H stretching vibrations, was more pronounced in the CS-CDs compared to the CS precursor, indicating an increased abundance of hydroxyl groups. The enhanced intensity of this band suggests the formation of oxygen-containing functional groups during the hydrothermal carbonization process [12]. A distinct peak at 1541–1548 cm^−1^, assigned to amide II vibrations, exhibited greater intensity in the CS-CDs than in the precursor [18]. This increase may be associated with decarboxylation and oxidation reactions occurring during hydrothermal treatment, leading to the formation of carbonyl-containing structures [4].

Additionally, the peaks observed at 1402–1421 cm^−1^ can be attributed to C-H bending or deformation vibrations of carbon frameworks, reflecting structural rearrangements within the carbon matrix. With increasing hydrothermal temperature, enhanced polymerization and crosslinking reactions likely contributed to the development of more complex carbon structures [4]. Meanwhile, the absorption band at 1018–1060 cm^−1^, corresponding to C-O-C stretching vibrations, gradually decreased in intensity with prolonged or intensified hydrothermal treatment. This reduction may be attributed to the progressive degradation of the chitosan backbone (cleavage of glycosidic linkages) and the dehydration, deamination, and carbonization of its glucosamine units during hydrothermal treatment [18]. Overall, the CS-CDs exhibited stronger and more defined characteristic peaks compared to the CS precursor, indicating the enrichment of surface functional groups. These changes confirm the successful transformation of the precursor into functionalized carbon dots through the hydrothermal process. A limitation of this study is that the fluorescence quantum yield of carbon dots was not determined. Since the present work focused primarily on their functional properties for food applications, future studies should quantify the quantum yield to allow a more complete characterization and comparison with other carbon dots.

#### 2.1.6. Antioxidant Properties

**Figure 7:**
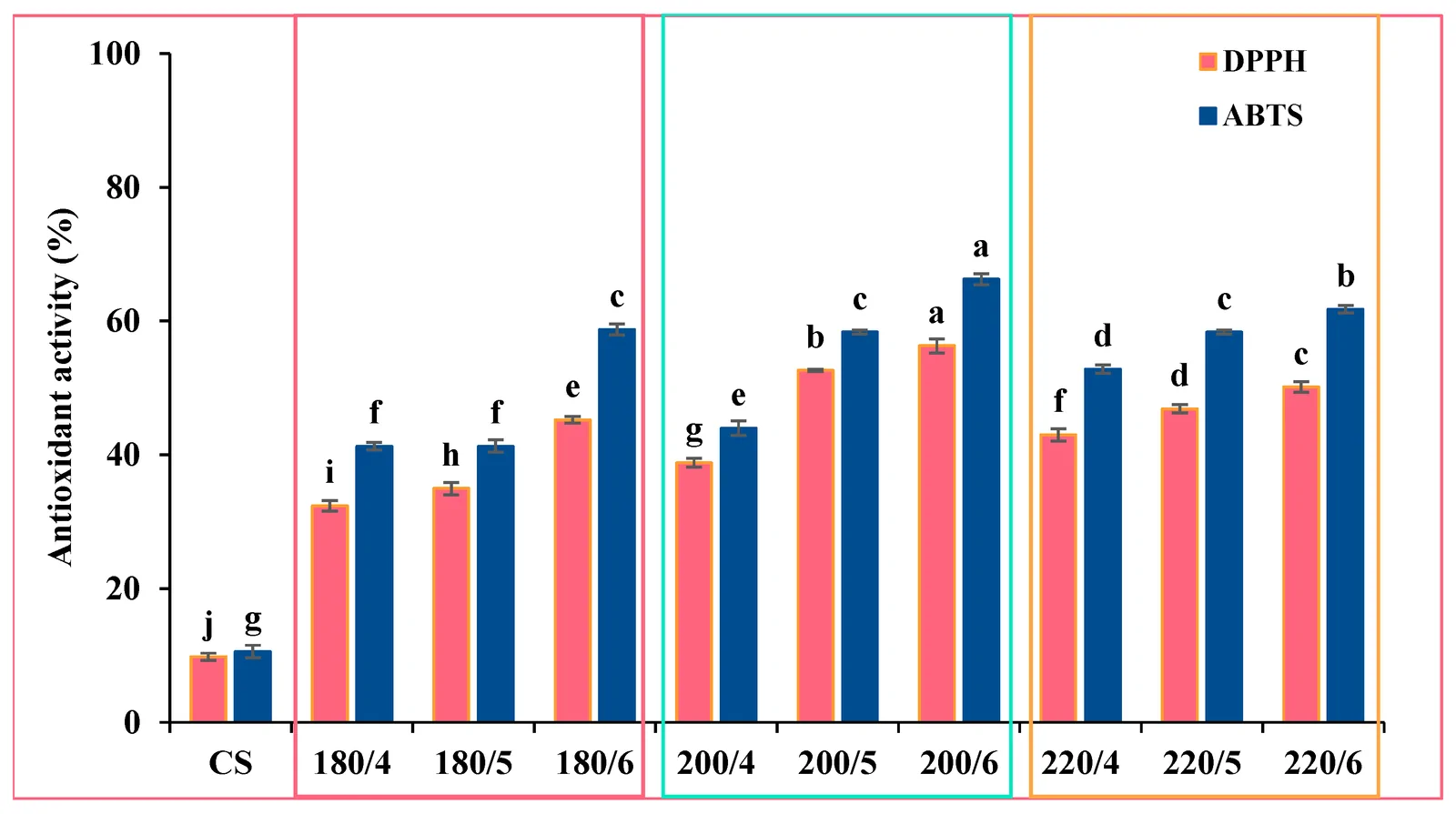
Antioxidant activity of CDs measured by ABTS• and DPPH• scavenging activities. Values are expressed as mean ± SD (n = 3). Different lowercase superscripts indicate significant differences (*p* < 0.05).

Antioxidant activity is a key indicator for evaluating the ability of compounds to scavenge or neutralize free radicals. In this study, the antioxidant capacity of CS-CDs was assessed using ABTS and DPPH radical scavenging assays, as shown in Figure 7. At a constant hydrothermal temperature, both ABTS• and DPPH• scavenging activities of the CS-CDs increased with prolonged reaction time. Overall, the ABTS assay demonstrated higher radical scavenging activity than the DPPH assay. The superior antioxidant performance observed in the ABTS assay can be attributed to the hydrophilic nature and excellent dispersion of CDs in aqueous systems, which enhances their interaction with ABTS radicals. Furthermore, the ABTS assay is capable of detecting both hydrophilic and lipophilic antioxidants, indicating the broad-spectrum radical scavenging potential of CS-CDs [15]. In contrast, the relatively lower scavenging activity observed in the DPPH assay may result from limited interaction between CDs and DPPH radicals in methanolic media [19].

CS-CDs synthesized at 200 °C for 6 h showed the highest activity in both assays, with scavenging of 56.29 ± 1.05% (DPPH) and 66.25 ± 0.82% (ABTS), which was significantly higher than that of the other conditions (*p* < 0.05; Figure 7) and about 5.7- and 6.3-fold higher than the CS precursor (9.80 ± 0.55% and 10.59 ± 0.93%). The enhanced antioxidant activity of CS-CDs is likely associated with the abundance of surface functional groups capable of donating electrons or hydrogen atoms to neutralize free radicals [13]. Functional groups such as hydroxyl (–OH), carboxyl (–COOH), and amino (–NH_2_) groups are known to play a crucial role in radical scavenging through hydrogen atom transfer mechanisms [20]. The presence of hydroxyl (O-H, C-O-H), carboxyl (C=O), amino (N-H), and ether (C-O-C) groups on the CS-CDs surface was consistent with the FTIR spectra (Figure 6). Overall, these results suggest that CS-CDs, particularly those synthesized at 200 °C for 6 h, possess strong antioxidant properties. Such characteristics are highly beneficial for potential applications in inhibiting lipid oxidation in food systems, as well as in biomedical fields.

#### 2.1.7. Antimicrobial Properties

**Figure 8:**
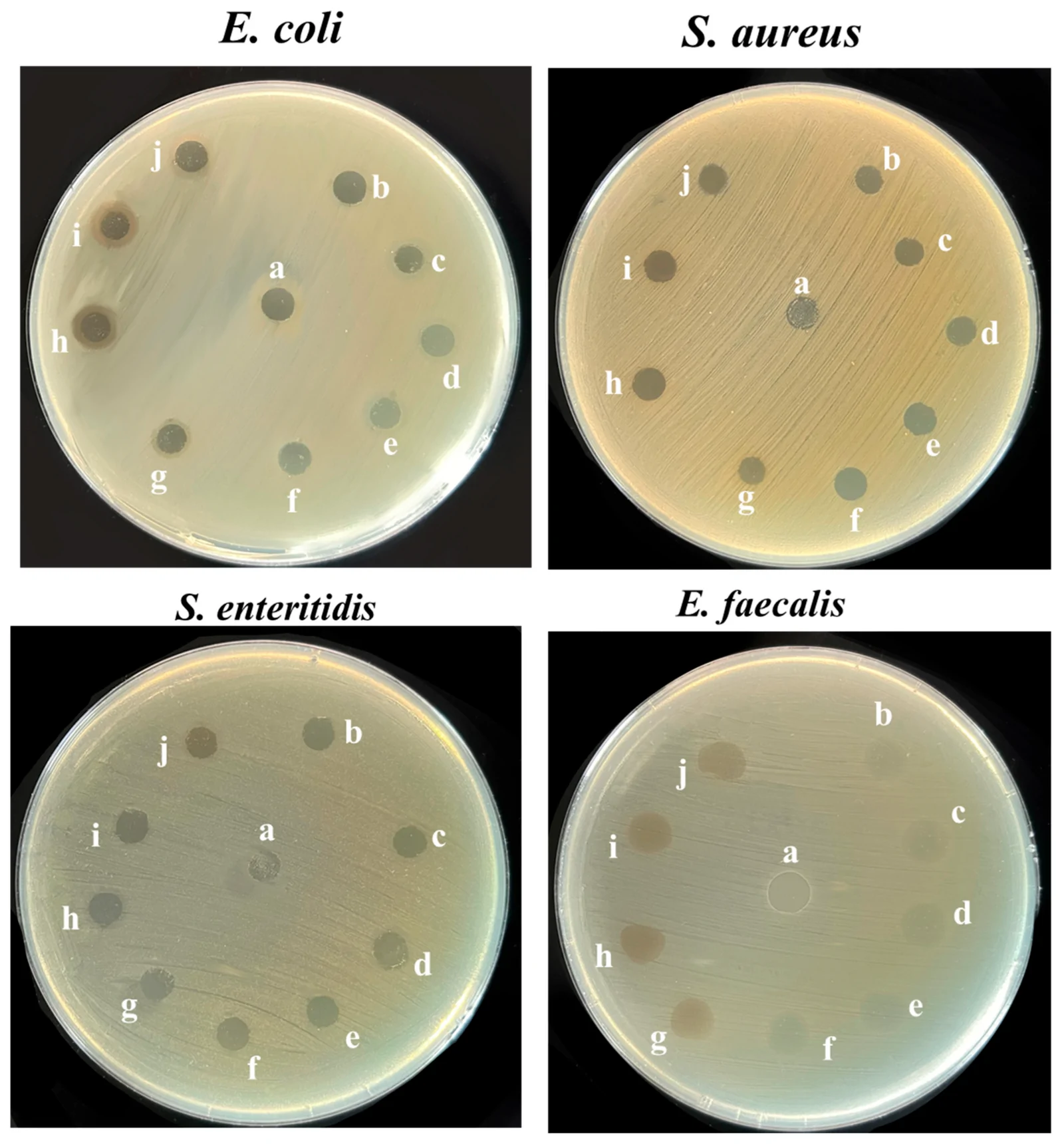
Antibacterial activity of CS-CDs determined by the disk diffusion method. Sterile paper disks (6 mm) loaded with 10 µL of each solution were placed on the inoculated agar and removed before photographing. (a: Chitosan, b: 180 °C for 4 h, c: 180 °C for 5 h, d: 180 °C for 6 h, e: 200 °C for 4 h, f: 200 °C for 5 h, g: 200 °C for 6 h, h: 220 °C for 4 h, i: 220 °C for 5 h, j: 220 °C for 6 h).

The antibacterial activity of CS and CS-CDs synthesized under different conditions was evaluated against four common foodborne pathogens (*S. aureus*, *E. faecalis*, *S. enteritidis*, and *E. coli*) using disk diffusion methods (Figure 8). The CS precursor showed clear antibacterial activity against *E. coli* and *S. aureus* but no inhibitory effect against *S. enteritidis* and *E. faecalis*. In contrast, CS-CDs exhibited broader antibacterial activity, inhibiting the growth of all four foodborne pathogens, except that CS-CDs synthesized at 180 showed no inhibitory activity against *E. faecalis*, whereas those synthesized at 200 °C exhibited only weak and partial inhibition. Apart from this, the differences in inhibition zones among the synthesis conditions were small by visual inspection and were not quantified. These assays were therefore used to demonstrate the presence or absence of antibacterial activity rather than to rank the synthesis conditions. Similar responses may be attributed to the common chitosan precursor, similar particle sizes, and comparable surface functional groups of the CS-CDs, as well as the limited sensitivity of agar diffusion methods to small differences in activity.

The antibacterial activity of CS-CDs may involve multiple synergistic mechanisms. First, electrostatic interactions between the positively charged surface of CS-CDs and the negatively charged bacterial cell membranes may promote adhesion and membrane disruption, increasing membrane permeability and allowing CS-CDs to penetrate into the cells. This may cause leakage of essential cellular components, including electrolytes and cytoplasmic contents, ultimately resulting in cell death [21]. In addition, the surface amino groups identified by FTIR are consistent with the proposed electrostatic and intracellular interactions. In particular, amine groups may interact with intracellular biomolecules such as nucleic acids, leading to DNA damage and disruption of cellular functions [21]. Such interactions may also promote the generation of reactive oxygen species (ROS), which induce oxidative stress and damage proteins, lipids, and other essential biomolecules; sulfur-derived species have also been suggested to react with membrane lipids, leading to lipid degradation, increased membrane permeability, and release of intracellular components [19]. Moreover, the nanoscale size of CS-CDs may facilitate their penetration into bacterial cells, where they can interfere with metabolic processes and normal cellular function [1]. Overall, these combined effects, including membrane disruption, oxidative stress, intracellular interactions, and size-dependent cellular penetration, may explain the antibacterial activity of CS-CDs observed in this study, although these mechanisms were not directly investigated here.

### 2.2. Fabrication of Gelatin Capsule Waste/Gum Arabic Composite Films

#### 2.2.1. Colorimetric Property of Gel/GA/CD Films

**Figure 9:**
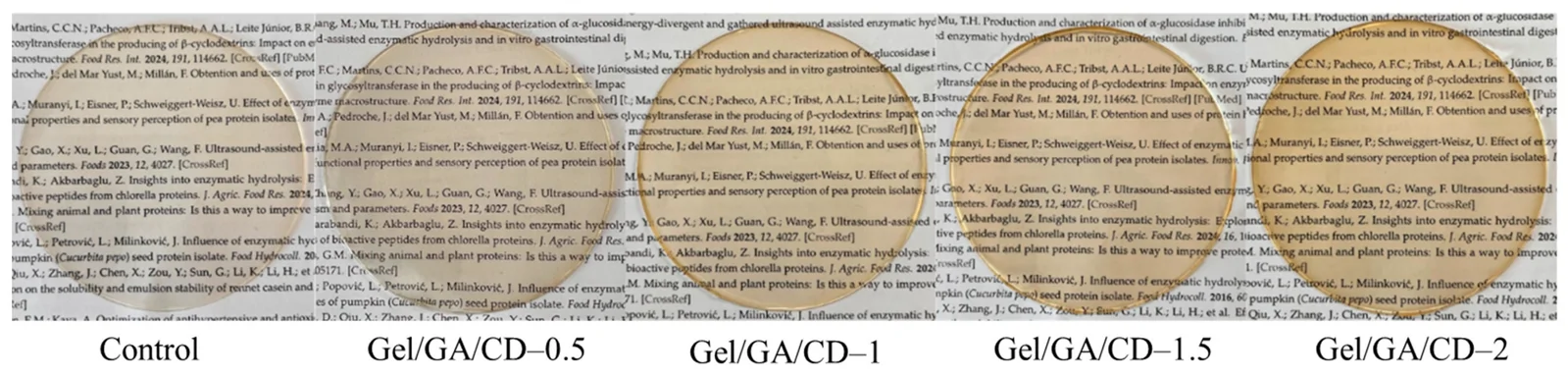
Appearance of Gelatin/Gum Arabic films incorporated with CS-CDs at different concentrations (0–2%) under daylight.

Carbon dots synthesized under optimal conditions (the condition with the highest antioxidant activity in both DPPH and ABTS assays was selected; 200 °C for 6 h) were selected for incorporation into Gel/GA/CD films at different levels (0, 0.5, 1, 1.5, or 2%). The visual appearance of films with and without CS-CDs is presented in Figure 9. The control film (without CS-CDs) exhibited higher transparency, which is characteristic of gelatin-based films [22]. Upon incorporation of CS-CDs, the films developed a yellowish tint, likely due to the intrinsic light brown color of the carbon dots. Moreover, the Gel/GA/CD films became progressively darker with increasing CS-CD concentration. This change in appearance indicates that the incorporation of CDs as fillers within the polymer matrix significantly affected the optical properties, particularly reducing film transparency.

The color parameters (L*, a*, and b*) of the different films are presented in Table 1. The incorporation of CS-CDs significantly decreased the lightness (L*) and redness (a*), while increasing the yellowness (b*) of the Gel/GA films (*p* < 0.05). These changes can be attributed to the inherent brownish coloration of the CS-CDs. This finding is consistent with previous reports, which indicated that the increase in b* values was associated with the incorporation of yellowish-brown CDs into biopolymeric films [23]. In general, the addition of active compounds, particularly those containing pigments, can alter the optical properties of films [24]. Therefore, the presence of CS-CDs had a direct impact on the visual appearance of the Gel/GA films, especially by enhancing their yellowness.

#### 2.2.2. Thickness of Gel/GA/CD Films

The thickness of all films is presented in Table 2. The incorporation of CS-CDs resulted in a slight but significant increase in film thickness (*p* < 0.05). This effect may be attributed to the presence of CDs, which could interfere with the intermolecular interactions and molecular alignment of the polymer chains, leading to a less ordered film matrix. Such structural disruption likely promoted the formation of a more heterogeneous and protruded network within the films, thereby contributing to the increased film thickness [23]. In this study, the thickness of the Gel/GA/CD-0.5 film increased by approximately 1.10-fold and further increased to about 1.20-fold with higher CS-CD concentrations (up to 2%), compared to the control film. This trend may also be associated with enhanced interactions and improved alignment between gelatin, GA, and CS-CDs within the film matrix, which can contribute to the formation of a more compact and structured network [22]. These findings are consistent with previous studies reporting that the incorporation of carbon dots into biopolymeric films leads to increased thickness. For instance, the addition of carbon dots derived from natural sources has been shown to directly influence the thickness of gelatin-based composite films [24]. Similarly, higher thickness values have been reported in films containing CDs, confirming that CD concentration plays a crucial role in determining the structural properties of the resulting films [23]. Overall, the results indicate that the thickness of Gel/GA films is significantly influenced by the incorporation and concentration of CS-CDs, reflecting their impact on film structure and matrix organization.

#### 2.2.3. Mechanical Properties of Gel/GA/CD Films

The mechanical properties of films are critical for their practical application in food packaging, as sufficient strength and structural integrity are required to prevent breakage or leakage during handling, storage, and distribution [23]. The neat Gel/GA film (control) exhibited good flexibility, with a tensile strength (TS) of 2.20 MPa and an elongation at break (EAB) of 41.67% (Table 2). Upon incorporation of CS-CDs, TS increased significantly (*p* < 0.05) by up to 90.5% (at 2.0% CS-CDs), whereas EAB decreased significantly (*p* < 0.05) by up to 30.0% relative to the control. These results indicate that the addition of CS-CDs enhanced film strength while reducing flexibility. Similar trends have been reported in previous studies, where the incorporation of carbon dots into the chitosan/gelatin film resulted in increased TS and decreased EAB [1]. The improvement in TS may be attributed to interactions between CS-CDs and the polymer matrix. As shown by FTIR (Section 2.1.5), CS-CDs possess abundant surface functional groups (–OH, –NH_2_, and –COOH), which may form hydrogen bonds and electrostatic interactions with the polar groups of gelatin and GA, as reported for similar carbon dot–biopolymer systems [1]. Such interactions may increase network compactness and restrict polymer chain mobility, resulting in higher TS and lower EAB. However, these interactions were not directly verified by FTIR of the composite films in this study, and further spectroscopic analysis is needed to confirm this mechanism. Overall, the results demonstrate that CS-CDs act as effective reinforcing agents in Gel/GA films, improving mechanical strength while reducing flexibility, which is advantageous for developing robust active packaging materials.

#### 2.2.4. Moisture Content and Water Solubility of Gel/GA/CD Films

The moisture content and water solubility of the films are presented in Table 2. Moisture content reflects the amount of water retained within the film matrix and provides insight into the film’s stability under varying humidity conditions [25]. The moisture content of the film decreased markedly from 22.37 ± 0.21% (control) to 7.28 ± 0.40% with 0.5% CS-CDs, a reduction of about 67%, and was lowest at 1% CS-CDs (6.82 ± 0.48%). At higher concentrations, the moisture content increased slightly to 7.68 ± 0.64% (1.5%) and 8.41 ± 0.29% (2%); the value at 2% was significantly higher than those at 0.5% and 1% (*p* < 0.05) but remained far below that of the control (Table 2). The pronounced initial decrease may be attributed to intermolecular interactions, including hydrogen bonding, between the surface functional groups of CS-CDs and the functional groups of Gelatin and Gum Arabic. These interactions may promote a more compact polymer network and reduce the accessibility of hydrophilic groups to water molecules, thereby limiting moisture content [26]. The slight increase at higher concentrations may reflect the contribution of the hydrophilic groups of the CDs themselves [26]. These explanations are tentative, as the interactions were not examined directly in this study. Similarly, the water solubility of the films decreased with the incorporation of CS-CDs. The control, Gel/GA/CD-0.5, and Gel/GA/CD-1.0 films showed relatively high solubility, whereas a significant reduction was observed at higher CS-CD concentrations, particularly at 1.5–2.0%. This behavior may be attributed to the formation of stronger interfacial interactions, such as hydrogen bonding and electrostatic interactions, between CS-CDs and the polymer matrix [1]. Overall, the incorporation of CS-CDs reduced the moisture content and water solubility of the Gel/GA films, indicating improved stability in humid and aqueous environments. This is beneficial for food packaging applications, whereas the reduced flexibility is a limitation that could be addressed by blending with other polymers, as discussed in the Conclusion. Other water-related properties, such as water vapor permeability, were not evaluated in this study.

#### 2.2.5. Antioxidant Properties of Gel/GA/CD Films

**Figure 10:**
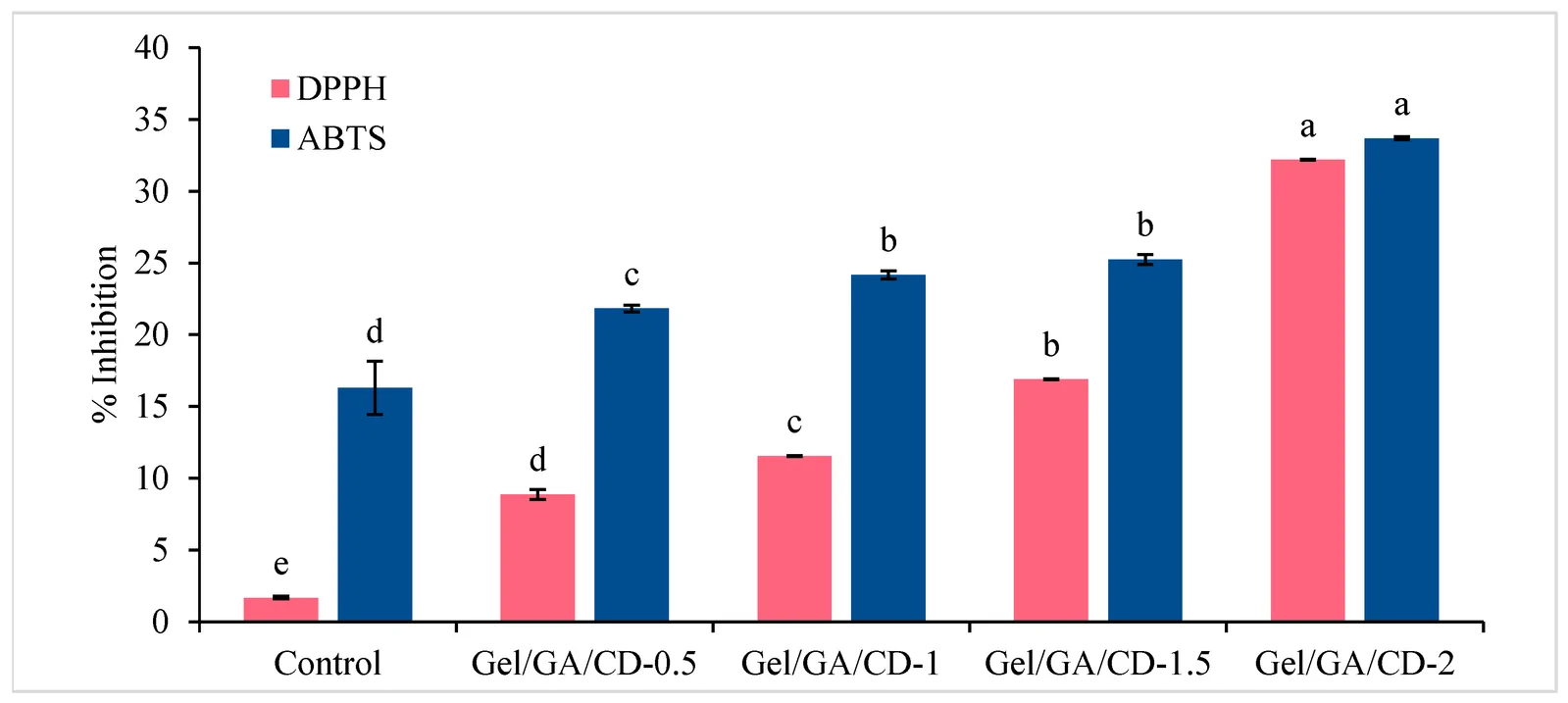
Antioxidant activity of Gelatin/Gum Arabic films incorporated with CS-CDs at different concentrations. Different lowercase superscripts indicate significant differences (*p* < 0.05). Gel/GA/CD: Gelatin/Gim Arabic/Carbon dots.

The antioxidant activity of the films, evaluated using DPPH and ABTS radical scavenging assays, is presented in Figure 10. The neat Gel/GA film (control) exhibited very low radical scavenging activity, with values of approximately 1.67% and 16.29% for DPPH and ABTS assays, respectively, indicating limited inherent antioxidant capacity. The incorporation of CS-CDs significantly enhanced the antioxidant performance of the films, with scavenging activities increasing to 8.87–32.18% (DPPH) and 21.82–33.68% (ABTS). Notably, the ABTS assay consistently showed higher scavenging activity than the DPPH assay. This difference can be attributed to the greater solubility and dispersion of both the biopolymer matrix and CS-CDs in the aqueous ABTS system compared to the methanolic DPPH system, which facilitates more effective interactions with ABTS radicals [24]. The improved antioxidant activity is primarily associated with the presence of electron- or hydrogen-donating functional groups, such as hydroxyl (-OH) and carboxyl (-COOH) groups, on the surface of CS-CDs. These functional groups can readily transfer hydrogen atoms or electrons to neutralize DPPH• and ABTS• radicals, thereby enhancing radical scavenging efficiency [5]. These findings are consistent with previous studies, which reported that the incorporation of carbon dots into biopolymer films significantly improves antioxidant properties, with higher scavenging activity generally observed against ABTS radicals compared to DPPH [27]. Overall, the results demonstrate that the incorporation of CS-CDs, particularly at higher concentrations (e.g., 2.0%), effectively enhances the antioxidant capacity of Gel/GA films. This improvement highlights their potential application as active packaging materials for retarding oxidative deterioration and extending the shelf life of food products.

#### 2.2.6. Antibacterial Efficiency of Gel/GA/CD Films

**Figure 11:**
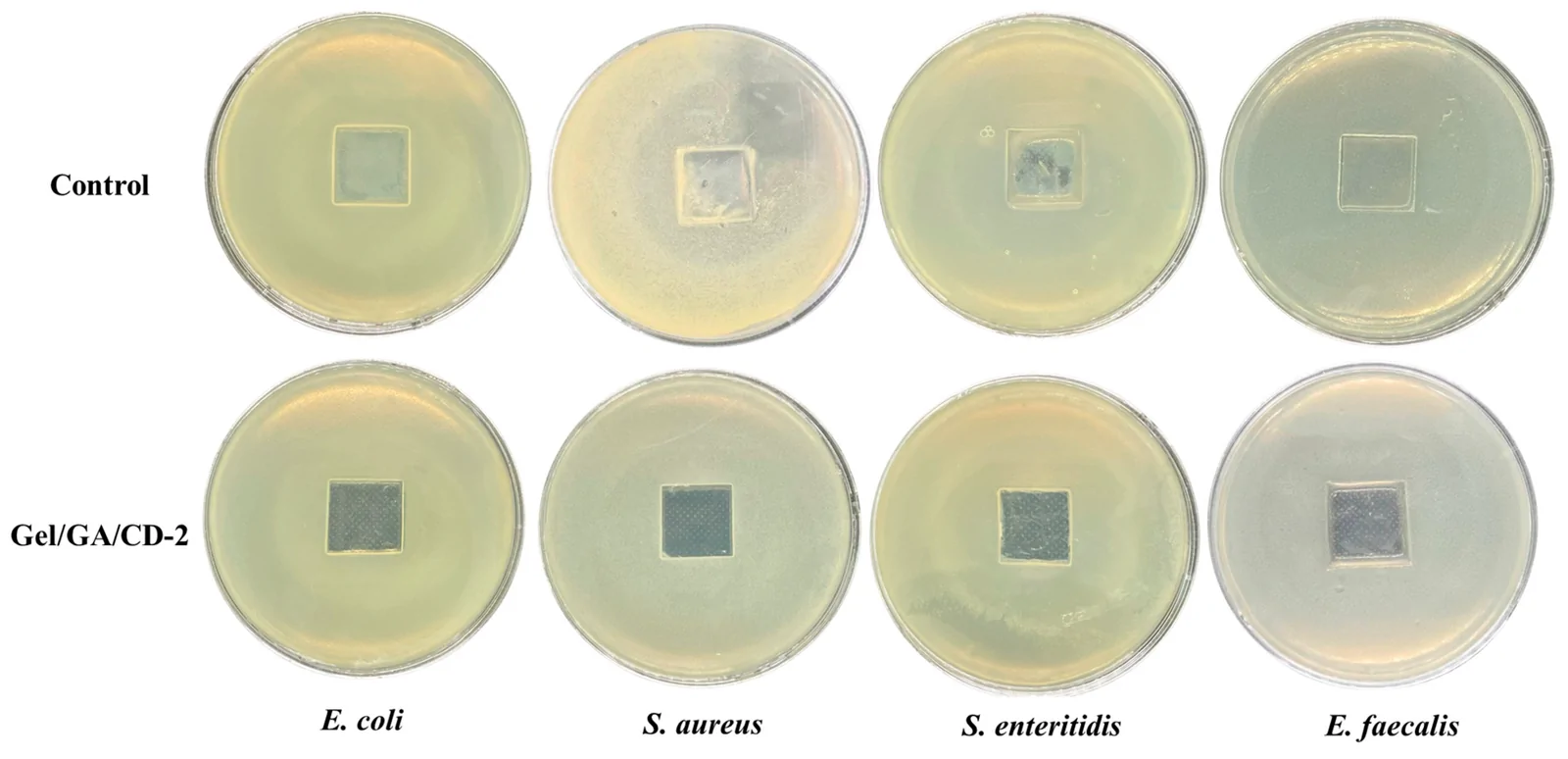
Antibacterial activity of the Gelatin/Gum Arabic-based films against *E. coli*, *S. aureus*, *S. enteritidis* and *E. faecalis*.

The antibacterial activity of the films was qualitatively assessed by observing the presence or absence of clear inhibition zones, which indicates the release of antibacterial agents from the films. As the inhibition zone size is influenced by film thickness and diffusion through the agar, this assay was used for screening rather than for comparing antibacterial efficiency among the films. Figure 11 shows the antibacterial activity of films containing CS-CDs against four foodborne pathogens (*E. coli*, *S. enteritidis*, *S. aureus* and *E. faecalis*). The Gel/GA film (control) exhibited no observable inhibition zones against any of the tested microorganisms, confirming the absence of inherent antibacterial activity. In contrast, the incorporation of 2% CS-CDs enhanced the antimicrobial performance of the films, resulting in effective inhibition of all tested pathogens except *E. faecalis*, for which inhibition was incomplete, as bacterial growth remained in some areas. These findings are consistent with Luan, Li, Lin, Lin, Zhu, Feng, Sun, Wu and Yang [5], who reported that the incorporation of 1.0% CDs from *Sargassum fusiforme* into biopolymer-based films markedly improves antimicrobial efficacy compared to control films. Similarly, they observed that films incorporated with carbon dots exhibited significant antibacterial activity against *L. monocytogenes* and *E. coli*, with complete inhibition after 6 h of exposure [1]. The improved antibacterial activity of Gel/GA/CD films may be primarily attributed to the intrinsic antimicrobial properties of CS-CDs and their gradual release from the polymer matrix into the surrounding medium. CDs are known to exert antibacterial effects through multiple mechanisms. Their small nanoscale size facilitates penetration through bacterial cell walls, enabling direct interaction with intracellular components and disruption of metabolic processes. Additionally, electrostatic interactions between positively charged CDs and negatively charged bacterial membranes promote membrane damage and increased permeability [23]. Furthermore, CDs can induce oxidative stress through the generation of reactive oxygen species (ROS), including singlet oxygen, superoxide anions, and hydroxyl radicals. These reactive species can damage essential biomolecules such as proteins, lipids, and nucleic acids, ultimately leading to cell dysfunction and lysis. CDs may also interfere with protein synthesis and DNA replication, further contributing to the breakdown of cellular machinery [1]. Overall, the results suggest that CS-CDs incorporated within the Gel/GA matrix can diffuse into the surrounding environment and effectively inhibit microbial growth. This highlights the potential of Gel/GA/CD films as active packaging materials with enhanced antimicrobial functionality for improving food safety and extending shelf life.

### 2.3. Application of Gelatin Capsule Waste/Gum Arabic Composite Films in Minced Fish

**Figure 12:**
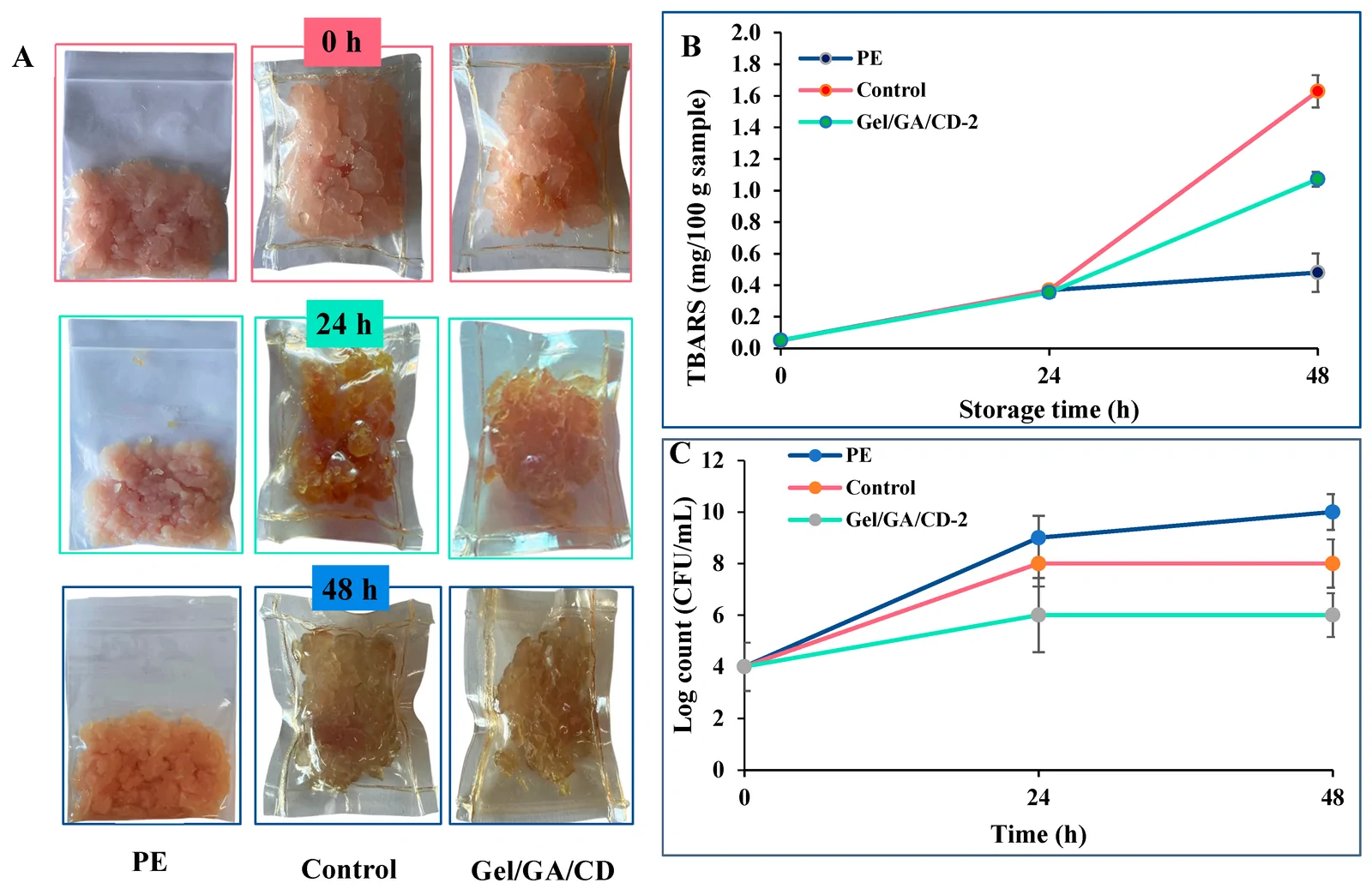
Appearance changes (**A**), TBARS (**B**) and bacterial growth (**C**) of minced fish wrapped with Polyethylene (PE), Gelatin/Gum Arabic film (Control) and Gelatin/Gum Arabic/CD film incorporated with 2.0% of CS-CDs (Gel/GA/CD-2) during storage at room temperature for 48 h.

A packaging test was conducted to evaluate the effectiveness of CS-CD-incorporated Gel/GA films for active food packaging applications. Minced fish samples were packaged using three different materials, i.e., polyethylene Ziplock bags (PE), control Gel/GA film (without CS-CDs), and Gel/GA/CD-2 film, and stored at room temperature (25 °C) for 48 h. Changes in product quality during storage are presented in Figure 12. As shown in Figure 12A, minced fish packaged in PE, as a commercial packaging reference, exhibited bleaching after 24 h and developed a greenish hue after 48 h, which is indicative of microbial spoilage [28]. Compared with the control, samples wrapped in Gel/GA/CD-2 films exhibited slower discoloration and better retention of their reddish color throughout storage. The improved color stability may be attributed to the combined antioxidant and antimicrobial properties of CS-CDs incorporated into the film matrix, which may have helped delay oxidative and microbial deterioration. The visual observations were consistent with lipid oxidation results (Figure 12B). Lipid oxidation, as measured by thiobarbituric acid reactive substances (TBARS), increased progressively in all treatments during storage, reflecting the accumulation of malondialdehyde, a secondary oxidation product [29]. At 48 h, minced fish packaged with Gel/GA/CD-2 showed significantly lower TBARS (1.07 mg/100 g) than that packaged with the CD-free Gel/GA film (1.63 mg/100 g), indicating that the incorporation of CS-CDs reduced lipid oxidation relative to the matched gelatin film. This may be related to the radical-scavenging activity of CS-CDs [13]. However, the PE-packaged sample showed lower TBARS (0.48 mg/100 g) at 48 h. This may reflect differences in package format, headspace, and light and oxygen exposure between PE bags and film wraps, which were not controlled in this study. Thus, Gel/GA/CD-2 did not outperform PE with respect to lipid oxidation under these conditions. Microbial growth during storage is presented in Figure 12C.

Minced fish, being rich in nutrients, provides a favorable environment for microbial proliferation. Generally, a total viable count exceeding 6 log CFU/g is considered indicative of spoilage and unfit for consumption [1]. As expected, samples packaged in PE showed rapid microbial growth, increasing from approximately 4 Log CFU/g to 9 Log CFU/g after 24 h and reaching 10 Log CFU/g after 48 h, which correlated with the observed spoilage characteristics. In the control Gel/GA film, microbial counts increased to 8 Log CFU/g after 24 h and remained at this level thereafter. In contrast, minced fish packaged with Gel/GA/CD-2 films maintained microbial counts at approximately 6 Log CFU/g throughout the storage period. Therefore, Gel/GA/CD-2 slowed microbial growth relative to the other treatments, but samples had reached the threshold by 24 h. This effect may be attributed to the release of CS-CDs from the film and their disruption of microbial cells [1], although release was not measured here. Overall, the incorporation of CS-CDs into Gel/GA films significantly improved their functional properties, including antioxidant and antibacterial activities of the gelatin film, and slowed spoilage of minced fish under an accelerated challenge (25 ± 3 °C, 48 h). They did not demonstrate shelf-life extension, which requires validation under refrigerated storage. The results demonstrate the strong potential of Gel/GA/CD-2 films as effective and sustainable active packaging materials for food preservation. In this study, the packaging test was conducted at room temperature (25 °C) for 48 h to accelerate spoilage and to evaluate the effect of the films within a short period. However, this condition does not represent real cold-chain storage of minced fish. Therefore, the efficacy of the Gel/GA/CD films under refrigerated conditions (e.g., 4 °C) over a longer storage period should be evaluated in future studies to assess their commercial applicability.

## 3. Materials and Methods

### 3.1. Materials

Chitosan (snow crab shells) and Gum Arabic were purchased from Bangkok Chemical Co., Ltd. (Bangkok, Thailand). Gelatin capsule waste (GCW) was obtained from Service Pack Manufacturing Co., Ltd. (Pathum Thani, Thailand). Methyl alcohol, glacial acetic acid, 2,2-diphenyl-1-picrylhydrazyl (DPPH), 2,2-azino-bis (3-ethylbenzothiazoline-6-sulphonic acid) diammonium salt (ABTS), and 2-thiobarbituric acid were purchased from Sigma Chemical Co. (St. Louis, MO, USA). *Escherichia coli* ATCC 25922, *Salmonella enteritidis* ATCC 13076, *Staphylococcus aureus* ATCC 25923 and *Enterococcus faecalis* ATCC 19433 were obtained from the Department of Fishery Products, Faculty of Fisheries, Kasetsart University, Thailand. Red Nile Tilapia was purchased from a local market in Bangkok (Thailand).

### 3.2. Synthesis of Chitosan-Derived Carbon Dots (CS-CDs)

Chitosan-derived carbon dots (CS-CDs) were synthesized via a hydrothermal method following a report by Sul, Ezati and Rhim [1] with slight modifications. Briefly, 0.5 g of chitosan powder was dispersed in 100 mL of 1% (*v*/*v*) acetic acid under continuous stirring until a homogeneous solution was obtained. The chitosan solution was then transferred into a Teflon-lined stainless-steel reactor. A series of experiments was conducted to determine the optimal synthesis conditions. The reaction temperature was varied at three levels (180, 200, and 220 °C), while the reaction time was adjusted to 4, 5, and 6 h. After completion of the reaction and subsequent cooling to room temperature, the resulting product was centrifuged at 10,000 rpm for 30 min to remove unreacted materials. The supernatant was then filtered through Whatman No. 1 filter paper to eliminate residual impurities. The filtrate was stored at 4 °C until further analysis. The obtained CS-CDs were designated according to the synthesis temperature and reaction time. Samples prepared at 180 °C for 4, 5, and 6 h were labeled as 180/4, 180/5, and 180/6, respectively. Similarly, CS-CDs synthesized at 200 °C were denoted as 200/4, 200/5, and 200/6, corresponding to reaction times of 4, 5, and 6 h, respectively. For the 220 °C condition, the samples were labeled as 220/4, 220/5, and 220/6 for reaction times of 4, 5, and 6 h, respectively.

### 3.3. Characterization of CS-CDs

#### 3.3.1. Determination of Optical Properties

The formation of CS-CDs was preliminarily verified by visual observation under both daylight and ultraviolet light. For observation under daylight, CS-CD samples were transferred into a cuvette, and images were recorded to document their characteristic brown coloration. For fluorescence analysis, the samples were placed in a UV transilluminator in a dark environment. Upon UV illumination, the CS-CDs exhibited a distinct blue fluorescence, which was photographed for documentation. This procedure provided qualitative confirmation of successful CS-CD synthesis based on their optical properties.

#### 3.3.2. Transmission Electron Microscope (TEM)

The morphology and elemental composition of the CS-CDs were characterized using transmission electron microscopy (TEM) (Hitachi HT7700, Tokyo, Japan), following the method of Thakur, Bains, Kumar, Goksen, Dhull, Ali, Kaushik, Iqbal and Chawla [12] with slight modifications. The instruments were operated at an accelerating voltage of 120 kV. For sample preparation, 10 μL of the liquid sample was dropped onto a carbon-coated copper grid and allowed to dry for approximately 10 min to form a thin film. The samples were then subjected to TEM analysis, and images were acquired using the TEM-Hitachi HT7700 system. Particle size was estimated from the TEM images using Image J version 1.54t, with the scale calibrated against the scale bar.

#### 3.3.3. UV–Vis Spectrophotometer

The UV–visible absorption spectra and UV–visible light transmittance spectra of the CS-CDs were recorded using a UV–Vis spectrophotometer (Thermo Fisher Scientific Inc., Waltham, MA, USA), following the method outlined by Murugan, Khan, Nilsuwan, Kim, Benjakul and Rhim [23] with slight modifications. The measurements were performed over a wavelength range of 200–800 nm, using deionized (DI) water as the reference. Prior to analysis, the crude CS-CD samples were diluted 100-fold with DI water to ensure uniform dispersion. The absorbance and transmittance spectra were then recorded and processed using UV-Probe software version 2.70.

#### 3.3.4. Fluorescence Spectroscopy

The fluorescence properties of the CS-CDs were analyzed using a fluorescence spectrophotometer (RF-6000, Shimadzu Corporation, Kyoto, Japan) at 25 °C with a 1 cm quartz cuvette. The CS-CDs crude samples were diluted 10^−4^-fold with DI water under identical instrument settings, and a water blank was subtracted. To determine the optimum excitation wavelength, excitation spectra were recorded from 200 to 600 nm (Figure 5A). Emission spectra were then recorded from 300 to 600 nm at excitation wavelengths of 290–390 nm in 10 nm increments (Figure 5B) [13].

#### 3.3.5. Fourier Transform Infrared Spectroscopy

The FTIR spectra of the samples were recorded using an FTIR spectrophotometer (Bruker Co., Ettlingen, Germany), following the method described by [13]. Freeze-dried CS-CDs were ground into a fine powder, pressed into pellets, and placed on the sample holder for analysis. The spectra were collected over the wavenumber range of 4000–400 cm^−1^ to identify functional groups and structural characteristics. Measurements were performed with 32 scans at a resolution of 4 cm^−1^. The obtained data were analyzed using OPUS 8.5 software (Bruker Optik GmbH, Ettlingen, Germany).

#### 3.3.6. Antioxidant Properties

The antioxidant activity of CS-CDs was evaluated using DPPH and ABTS radical scavenging assays [22]. Briefly, 800 μL of DPPH or ABTS working solution was mixed with 200 μL of the crude CS-CD solution, and the mixture was incubated at room temperature for 30 min in the dark. After incubation, the absorbance was measured at 537 nm for DPPH and 734 nm for ABTS using a microplate reader (SPECTROstar Nano, BMG LABTECH, Ortenberg, Germany). Solvent blanks (1% acetic acid or water, respectively, without sample) were subjected to the same procedure, and their absorbance was used for correction. The antioxidant activity was then calculated as follows:

(1) $$\mathrm{Antioxidant}\;\mathrm{activity}\;\left(\%\right)=\frac{A0-\mathrm{At}}{A0}\times 100$$

where *A*0 and *At* are the absorbance values of the control and CD samples, respectively.

#### 3.3.7. Antimicrobial Properties

The antimicrobial activity of CS-CDs was evaluated following the method described by Kumnerdsiri, Sanprasert, Praiboon, Seubsai, Sirisarn, Pongsetkul, Harnkarnsujarit, Rawdkuen, Karnjanapratum, Sai-Ut and Kingwascharapong [22], with slight modifications. Briefly, bacterial strains, including *S. aureus*, *E. faecalis*, *S. enteritidis*, and *E. coli*, were cultured overnight at 37 °C in Mueller–Hinton (MH) broth. The cultures were then adjusted to a concentration of approximately 10^7^ CFU/mL and uniformly swabbed onto MH agar plates in three directions to ensure even distribution. The crude samples were sterilized by filtration through a 0.22 μm hydrophilic PTFE membrane filter. Subsequently, sterile paper disks (6 mm) loaded with 10 µL of each solution were placed on the MH agar plates. The plates were then incubated at 37 °C in a 5% CO_2_ atmosphere for 24 h. Antimicrobial activity was assessed by observing the presence of clear inhibition zones on the bacterial lawn, indicating bacterial sensitivity to the samples.

### 3.4. Fabrication of Gelatin Capsule Waste/Gum Arabic Composite Films

The carbon dot-incorporated film was prepared as a proof of concept to demonstrate the potential of the carbon dots as functional additives for food packaging. Gelatin capsule waste/Gum Arabic/carbon dots composite (Gel/GA/CD) films were prepared following the method described by Kumnerdsiri, Sanprasert, Praiboon, Seubsai, Sirisarn, Pongsetkul, Harnkarnsujarit, Rawdkuen, Karnjanapratum, Sai-Ut and Kingwascharapong [22] with slight modifications. Briefly, 40 g of gelatin capsule waste was dissolved in 50 mL of distilled water and heated at 50 °C under continuous stirring for 30 min until completely dissolved. A stabilizing solution was prepared by dissolving 10 g of GA in 40 mL of distilled water, followed by heating at 60 °C with constant stirring until a homogeneous solution was obtained. The GA solution was then added to the gelatin solution and further mixed for 10 min to ensure uniformity. Subsequently, carbon dots, synthesized under optimal conditions (the condition with the highest antioxidant activity in both DPPH and ABTS assays was selected; 200 °C for 6 h), were incorporated into the film-forming solution at concentrations of 0, 0.5, 1.0, 1.5, and 2.0% (*w*/*v*). The mixtures were continuously stirred for 15 min to achieve uniform dispersion of the CDs. Finally, 10 g of each film-forming solution was cast onto Petri dishes and dried at room temperature (25 °C) until completely dry. The resulting films were peeled off and stored in polyethylene ziplock bags within a desiccator maintained at 50% relative humidity prior to further analysis. The Gel/GA/CD films were designated based on the concentration of CS-CDs incorporated into the film-forming solution. Samples containing 0.5, 1.0, 1.5, and 2.0% (*w*/*v*) CS-CDs were labeled as Gel/GA/CD-0.5, Gel/GA/CD-1, Gel/GA/CD-1.5, and Gel/GA/CD-2, respectively, while the film without added CDs was used as the control.

### 3.5. Film Characterization

#### 3.5.1. Colorimetric Property of Gel/GA/CD Films

The color characteristics of Gel/GA/CD films were determined using a CIE colorimeter (ColorFlex, HunterLab, Reston, VA, USA). The color parameters were expressed as L* (lightness), a* (redness/greenness), and b* (yellowness/blueness) values. Measurements were performed at ten randomly selected locations on each film sample, and the mean values were calculated and used for subsequent analysis.

#### 3.5.2. Thickness of Gel/GA/CD Films

A micrometer (Mitutoyo, Model ID-C112PM, Serial No. 00320, Mitutoyo Corp., Kawasaki, Japan) was used to measure the film’s thickness. Ten randomly selected points on each film were measured in order to determine the average thickness.

#### 3.5.3. Mechanical Properties of Gel/GA/CD Films

Tensile strength (TS) and elongation at break (EAB) were determined using a Universal Testing Machine (Lloyd Instruments, Hampshire, UK) in accordance with the ASTM standard method. Film samples were cut into strips measuring 10 × 50 mm. The initial grip separation was set at 30 mm, and the crosshead speed was maintained at 30 mm/min. TS and EAB values were calculated using Equations (2) and (3)

(2) $$\mathrm{TS}=\frac{F}{A}$$

(3) $$\mathrm{EAB}(\%)=\frac{L1-L0}{L0}\times 100$$

where F signifies the maximum tensile force at fracture (N), A denotes the cross-sectional area of the film (mm^2^), L0 represents the initial grip separation (30 mm), and L1 indicates the final grip separation post-fracture (mm). Since 1 N/mm^2^ = 1 MPa, the result is expressed in MPa [22].

#### 3.5.4. Moisture Content and Water Solubility of Gel/GA/CD Films

Film samples (2 × 2 cm) were initially weighed (W_0_) and dried in a hot-air oven (Model WTB.1, Memmert, Schwabach, Germany) at 105 °C for 24 h. The dried samples were then reweighed to obtain W_1_. Subsequently, the films were immersed in distilled water (30 mL) at room temperature for 24 h. After immersion, the mixture was filtered through filter paper (Whatman No. 1) to recover the undissolved fraction, which was then dried again at 105 °C for 24 h and weighed (W_2_). Material passing through the filter (dissolved or finely dispersed) is accounted for by the difference between W1 and W2. The moisture content (MC) and water solubility (WS) of the films were calculated using Equations (4) and (5).

(4) $$\mathrm{MC}(\%)=\frac{W_{0}-W_{1}}{W_{0}}\times 100$$

(5) $$\mathrm{WS}\left(\%\right)=\frac{W_{1}-W_{2}}{W_{1}}\times 100$$

#### 3.5.5. Antioxidant Properties of Gel/GA/CD Films

Film samples (0.5 g) were dissolved in 5 mL of distilled water at 50 °C for 10 min until the solutions were clear with no visible precipitate (100 mg film/mL). The solutions were vortexed before each aliquot was taken for analysis, and the control film was treated in the same way. Based on the film formulation, 0.5 g of film contained approximately 4.95, 9.80, 14.56, and 19.23 mg of crude CS-CDs for the 0.5, 1.0, 1.5, and 2.0% (*w*/*v*) films, corresponding to 0.99, 1.96, 2.91, and 3.85 mg/mL in the 5 mL solution before dilution in the assay. The antioxidant activity of the prepared solutions was subsequently determined using DPPH and ABTS radical scavenging assays, following the procedure described in Section 3.3.6.

#### 3.5.6. Antibacterial Efficiency of Gel/GA/CD Films

The Gel/GA/CD-2 film, which exhibited the most favorable physicochemical and functional properties, was selected for evaluation of antibacterial activity in comparison with the control film (without added CDs). Film samples were cut into square pieces (2 × 2 cm) and sterilized under ultraviolet irradiation for 15 min on each side. The antimicrobial activity was qualitatively assessed by observing the presence or absence of clear inhibition zones surrounding the film samples, following the procedure described in Section 3.3.7.

### 3.6. Application of Gelatin Capsule Waste/Gum Arabic Composite Films in Minced Fish

The Gel/GA/CD-2 film was selected for application in food packaging. The packaging performance of polyethylene ziplock bags (PE), the control film (without added CDs), and the Gel/GA/CD-2 film was evaluated using a minced fish model. Minced fish samples (5 g) were individually wrapped with polyethylene ziplock bags (PE), control film (E0), or Gel/GA/CD-2 film (5 × 5 cm^2^). The packaged samples were then stored at room temperature (25 ± 3 °C) for 48 h to simulate accelerated spoilage conditions. Changes in visual appearance, surface color, moisture content, lipid oxidation, and microbial growth were monitored throughout the storage period.

Lipid oxidation during storage was evaluated using the thiobarbituric acid reactive substances (TBARS) assay, following the method of Rodsamai et al. [30] with slight modifications. Briefly, 0.25 g of the sample was mixed with 1.25 mL of thiobarbituric acid (TBA) reagent, consisting of 15% (*w*/*v*) trichloroacetic acid and 0.375% (*w*/*v*) TBA prepared in 0.25 M HCl. The mixture was homogenized and then heated in a boiling water bath for 15 min. After heating, the samples were immediately cooled in an ice bath and centrifuged at 3000× *g* for 10 min. The absorbance of the resulting supernatant was measured at 532 nm using a spectrophotometer. A standard curve was prepared using 1,1,3,3-tetraethoxypropane, and the results were expressed accordingly.

Microbial quality was assessed using the total viable count (TVC) method. Minced fish samples (5 g) were aseptically transferred into 45 mL of 0.1% peptone water and homogenized using a stomacher for 180 s. Serial ten-fold dilutions were subsequently prepared, and 0.1 mL aliquots from appropriate dilutions were spread-plated in triplicate. The total viable count was determined on Tryptic Soy Agar (TSA) after incubation at 37 °C for 24 h. Microbial counts were expressed as log CFU/g of sample [1]. The counts represent only the organisms recoverable under these conditions, and psychrotrophic, anaerobic, and other organisms were not assessed.

### 3.7. Statistical Analysis

The results are expressed as mean ± standard deviation. Statistical analysis was performed using analysis of variance (ANOVA), followed by Duncan’s multiple range test to compare mean differences. All analyses were carried out using SPSS software (version 25 for Windows; SPSS Inc., Chicago, IL, USA), and statistical significance was set at *p* < 0.05.

## 4. Conclusions

This study successfully demonstrated the synthesis of carbon dots (CDs) derived from chitosan using a hydrothermal method. The obtained CS-CDs exhibited desirable physicochemical properties, including a particle size below 10 nm, strong fluorescence emission, high stability, and broad UV–Vis absorption characteristics. Notably, CS-CDs synthesized at 200 °C for 6 h showed antibacterial and antioxidant activities. Incorporation of CS-CDs into Gel/GA film resulted in a homogeneous and relatively transparent composite with enhanced UV-blocking capability. The Gel/GA/CD films exhibited significant antioxidant and antimicrobial properties, with the formulation containing 2% CDs demonstrating the most effective performance. Application of the Gel/GA/CD-2 film for packaging minced fish effectively preserved product quality. Discoloration was reduced, while lipid oxidation and microbial growth were significantly inhibited compared to the control. These findings highlight the potential of CS-CD-incorporated biopolymer films as active packaging materials for food preservation. Overall, this work presents a sustainable approach for producing high-quality fluorescent carbon dots from chitosan with multifunctional properties, including antimicrobial, antioxidant, and UV-protective effects. Future research should focus on improving the mechanical properties of gelatin capsule waste-based films by blending them with other polymers, aiming to achieve performance comparable to that of conventional commercial packaging and to further extend food shelf life. Beyond active packaging, the multifunctional properties of CS-CDs suggest their potential use in other fields, such as biomedical scaffolds, wound healing, drug delivery systems, and fluorescent sensors. Nevertheless, further studies on biocompatibility and drug release behavior are required to validate these applications. In addition, future studies should evaluate the preservation efficacy of the films under the actual storage and distribution conditions of the target food (e.g., refrigerated minced fish over extended storage periods), which are more relevant to commercial applications.

## Figures and Tables

**Table 1:** Color of Gelatin/Gum Arabic-based films incorporated with various concentrations (0–2%) of CS-CDs.

Sample	Lightness (L*)	Redness (a*)	Yellowness (b*)
**Control**	88.46 ± 0.02 ^a^	1.16 ± 0.01 ^a^	−0.60 ± 0.01 ^e^
**Gel/GA/CD–0.5**	87.78 ± 0.08 ^b^	0.63 ± 0.01 ^b^	3.65 ± 0.01 ^d^
**Gel/GA/CD–1**	86.44 ± 0.95 ^c^	0.24 ± 0.01 ^c^	7.88 ± 0.01 ^b^
**Gel/GA/CD–1.5**	86.35 ± 0.04 ^c^	0.34 ± 0.01 ^d^	7.45 ± 0.01 ^c^
**Gel/GA/CD–2**	84.85 ± 0.02 ^d^	0.12 ± 0.01 ^e^	12.35 ± 0.01 ^a^

Values are expressed as mean ± SD. Different lowercase superscripts within the same column indicate significant differences (*p* < 0.05). Gel/GA/CD: Gelatin/Gum Arabic/carbon dots.

**Table 2:** The mechanical properties, moisture, and solubility of the Gel/GA/CD films.

Sample	Thickness (mm)	TS (MPa)	EAB (%)	Moisture (%)	Solubility
**Control**	0.29 ± 0.02 ^c^	2.20 ± 0.92 ^d^	41.67 ± 4.30 ^a^	22.37 ± 0.21 ^a^	93.56 ± 0.07 ^a^
**Gel/GA/CD–0.5**	0.32 ± 0.01 ^b^	3.09 ± 0.49 ^c^	38.34 ± 1.92 ^ab^	7.28 ± 0.40 ^cd^	90.68 ± 0.90 ^a^
**Gel/GA/CD–1**	0.34 ± 0.01 ^a^	3.18 ± 0.15 ^bc^	37.50 ± 1.67 ^ab^	6.82 ± 0.48 ^d^	93.17 ± 0.40 ^a^
**Gel/GA/CD–1.5**	0.34 ± 0.02 ^a^	3.83 ± 0.86 ^ab^	34.17 ± 5.00 ^bc^	7.68 ± 0.64 ^bc^	62.82 ± 4.02 ^b^
**Gel/GA/CD–2**	0.35 ± 0.02 ^a^	4.19 ± 0.97 ^a^	29.17 ± 3.19 ^c^	8.41 ± 0.29 ^b^	70.18 ± 10.59 ^b^

Values are expressed as mean ± SD. Different lowercase superscripts within the same column indicate significant differences (*p* < 0.05). Gel/GA/CD: Gelatin/Gim Arabic/Carbon dots.

## Data Availability

The original contributions presented in this study are included in the article. Further inquiries can be directed to the corresponding author.
